# FraudMiner: A Novel Credit Card Fraud Detection Model Based on Frequent Itemset Mining

**DOI:** 10.1155/2014/252797

**Published:** 2014-09-11

**Authors:** K. R. Seeja, Masoumeh Zareapoor

**Affiliations:** Department of Computer Science, Jamia Hamdard University, New Delhi 110062, India

## Abstract

This paper proposes an intelligent credit card fraud detection model for detecting fraud from highly imbalanced and anonymous credit card transaction datasets. The class imbalance problem is handled by finding legal as well as fraud transaction patterns for each customer by using *frequent itemset mining.* A matching algorithm is also proposed to find to which pattern (legal or fraud) the incoming transaction of a particular customer is closer and a decision is made accordingly. In order to handle the anonymous nature of the data, no preference is given to any of the attributes and each attribute is considered equally for finding the patterns. The performance evaluation of the proposed model is done on UCSD Data Mining Contest 2009 Dataset (anonymous and imbalanced) and it is found that the proposed model has very high fraud detection rate, balanced classification rate, Matthews correlation coefficient, and very less false alarm rate than other state-of-the-art classifiers.

## 1. Introduction

With the advent of communications techniques, e-commerce as well as online payment transactions are increasing day by day. Along with this financial frauds associated with these transactions are also intensifying which result in loss of billions of dollars every year globally. Among the various financial frauds, credit card fraud is the most old, common, and dangerous one due to its widespread usage because of the convenience it offers to the customer. Also the various types of benefits like cash back, reward points, interest-free credit, discount offers on purchases made at selected stores, and so forth tempt the customers to use credit card instead of cash for their purchases. According to Kount, one of the top five fraud detection consultants revealed by http:\\www.topcreditcardprocessors.com in the month of August 2013, 40% of the total financial fraud is related to credit card and the loss of amount due to credit card fraud worldwide is $5.55 billion. Fraudster gets access to credit card information in many ways. According to a latest report by CBC News (http:\\www.huffingtonpost.ca/2013/04/24/smartphones-steal-credit-card-%20data_n_3148170.html), smart phones are used to skim credit card data easily with a free Google application. However, fraud is becoming increasingly more complex and financial institutions are under increasing regulatory and compliance pressures. In order to combat these frauds, banks need more sophisticated techniques of fraud detection. The major problem for e-commerce business today is that fraudulent transactions appear more and more like legitimate ones [1] and simple pattern matching techniques are not efficient to detect fraud. Moreover, the credit card transaction datasets are highly imbalanced and the legal and fraud transactions vary at least hundred times [2]. Generally, in real case, 99% of the transactions are legal while only 1% of them are fraud [3].

Fraud detection is generally viewed as a data mining classification problem, where the objective is to correctly classify the credit card transactions as legitimate or fraudulent. Even though fraud detection has a long history, not that much research has appeared in this area. The reason is the unavailability of real world data on which researchers can perform experiments since banks are not ready to reveal their sensitive customer transaction data due to privacy reasons. Moreover, they used to change the field names so that the researcher would not get any idea about actual fields.

Due to this scarcity of real dataset, not many fraud detection models have been developed and described in the academic literature, and even fewer are known to have been implemented in actual detection systems. Still we can find some successful applications of various data mining techniques [4, 5] like outlier detection, self-organizing maps, neural network, Bayesian classifier, support vector machine, artificial immune system, fuzzy systems, genetic algorithm, K-nearest neighbor, and hidden Markov model in fraud detection.

There are many review papers describing the different types of frauds and different fraud detection techniques [6–11]. CardWatch [12] is a neural network based credit card fraud detection which trains a neural network with the past data of particular customer spending behavior and the authors tested their software on synthetically generated data. Ghosh and Reilly [13] developed a multilayer feedforward neural network based fraud detection model for Mellon Bank. Self-organizing map neural network [14–16] is a clustering technique used by many researchers to detect credit card fraud based on customer behaviour. Duman and Ozcelik [17] suggested a combination of genetic algorithm and scatter search for improving the credit card fraud detection and the experimental results show a performance improvement of 200%. Maes et al. [18] have performed experiments using Bayesian belief networks and artificial neural networks on credit card fraud detection. They found that Bayesian network has better fraud detection capability than artificial neural network. Srivastava et al. [19] proposed a credit card fraud detection model using hidden Markov model. In their research they trained the HMM with the normal behavior of the customer and the incoming transaction is considered as fraudulent if it is not accepted by the HMM with high probability. Chan et al. [20] addressed the issues of skewed distribution of credit card data and nonuniform cost by using a distributed data mining model. Seyedhossein and Hashemi [21] proposed a fraud detection method which extracts the inherent pattern from a credit card time series and uses this pattern for earlier fraud detection. Sánchez et al. [22] suggested and demonstrated the use of fuzzy association rule mining in extracting knowledge useful for fraud detection from transactional credit card databases. Syeda et al. [23] suggested a parallel processing environment for fast training of neural network for fraud detection. Wong et al. [24] investigated the application of artificial immune system in credit card fraud detection even though it was a preliminary research. Lu and Ju [2] designed a class weighted support vector machine for classifying the imbalanced credit card transaction data. Panigrahi et al. [25] integrated three different approaches—rule-based filtering, Dempster-Shafer theory, and Bayesian learning—for reducing false alarms in credit card fraud detection. Jha et al. [26] employed transaction aggregation strategy to capture costumer buying behaviour prior to each transaction and used these aggregations to detect credit card fraud.

Most of the work found in the literature works on customer spending behaviour analysis and some of them use some derived attributes also. However, we could not find any research performed on anonymous credit card transaction dataset where the derived attribute concept fails. Thus, the objective of this research was to develop a credit card fraud detection model which can effectively detect frauds from imbalanced and anonymous dataset. In order to handle anonymous data, which is the nature of data generally banks provide due to security reasons, the proposed fraud detection model considered each attribute equally without giving preference to any attribute in the dataset. Also the proposed fraud detection model creates separate legal transaction pattern (costumer buying behaviour pattern) and fraud transaction pattern (fraudster behaviour pattern) for each customer and thus converted the imbalanced credit card transaction dataset into a balanced one to solve the problem of imbalance.

## 2. Material and Methods

### 2.1. Support Vector Machine

The support vector machines (SVM) are statistical learning techniques first introduced by Cortes and Vapnik (1995) and they have been found to be very successful in a variety of classification tasks [27]. Support vector machines are based on the conception of decision planes which define decision boundaries. A decision plane is one that separates between a set of different classes. Basically SVM classification algorithms tend to construct a hyperplane as the decision plane which does separate the samples into the two classes—positive and negative. The strength of SVMs comes from two main properties: kernel representation and margin optimization. Kernels, such as radial basis function (RBF) kernel, can be used to learn complex regions. This algorithm finds a special kind of linear model, the maximum margin hyperplane, and it classifies all training instances correctly by separating them into correct classes through a hyperplane. The maximum margin hyperplane is the one that gives the greatest separation between the classes. The instances that are nearest to the maximum margin hyperplane are called support vectors. There is always at least one support vector for each class, and often there are more. In credit card fraud detection, for each test instance, it determines if the test instance falls within the learned region. Then if a test instance falls within the learned region, it is declared as normal; else it is declared as anomalous. This model has been demonstrated to possess a higher accuracy and efficiency of credit card fraud detection compared with other algorithms. Even for multidimensions and continuous features SVMs are the one of first choice [27]. SVM methods require large training dataset sizes in order to achieve their maximum prediction accuracy.

### 2.2. K-Nearest Neighbor

The K-nearest neighbor (KNN) technique [28] is a simple algorithm which stores all available instances; then it classifies any new instances based on a similarity measure. The KNN algorithm is example of an instance based learner. In the* nearest neighbor *classification method, each new instance is compared with existing ones by using a distance metric, and the closest existing instance is used to assign the class to the new one [11]. Sometimes more than one nearest neighbor is used, and the majority class of the closest K neighbors is assigned to the new instance. Among the various credit card fraud detection methods, the KNN achieves consistently high performance, without a priori assumptions about the distributions from which the training examples are drawn. In the process of KNN, we classify any incoming transaction by calculating nearest point to new incoming transaction. If the nearest neighbor is fraudulent, then the transaction is classified as fraudulent and if the nearest neighbor is legal, then it is classified as legal.

### 2.3. Naïve Bayes

Naïve Bayes (NB) is a supervised machine learning method that uses a training dataset with known target classes to predict the future or any incoming instance's class value. Naïve Bayes classifier is noted as a powerful probabilistic method that exploits class information from training dataset to predict the class of future instances [18, 29]. Naïve Bayes method assumes that the presence or absence of any attribute of a class variable is not related to the presence or absence of any other attributes. This technique is named “*naïve*” because it naïvely assumes independence of the attributes [11]. The classification is done by applying “Bayes” rule to calculate the probability of the correct class. Despite their naïve design and oversimplified assumptions, Naïve Bayes classifiers have good performance in many complex real world datasets.

### 2.4. Random Forest

Random forest [30] is an ensemble of decision trees. The basic principle behind ensemble methods is that a group of “weak learners” can come together to form a “strong learner.” Random forests grow many decision trees. Here each individual decision tree is a “weak learner,” while all the decision trees taken together are a “strong learner.” When a new object is to be classified, it is run down in each of the trees in the forest. Each tree gives a classification output or “vote” for a class. The forest classifies the new object into the class having maximum votes. Random forests are fast and they can efficiently handle unbalanced and large databases with thousands of features.

### 2.5. FraudMiner

**Figure 1:**
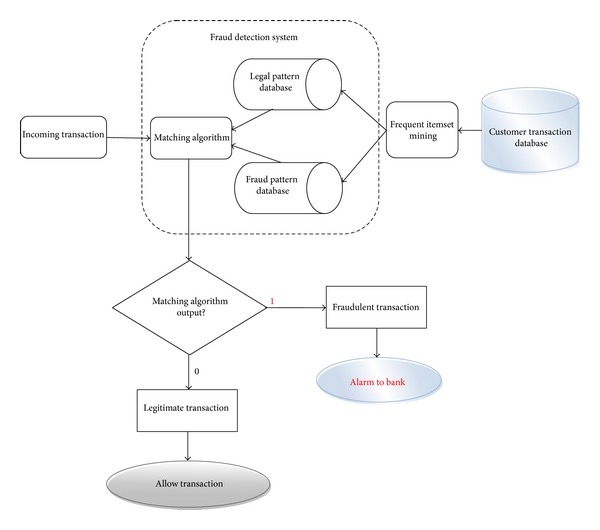
Proposed credit card fraud detection model.

The proposed fraud detection model (FraudMiner) is outlined in Figure 1. During the training phase, legal transaction pattern and fraud transaction pattern of each customer are created from their legal transactions and fraud transactions, respectively, by using frequent itemset mining. Then during the testing phase, the matching algorithm detects to which pattern the incoming transaction matches more. If the incoming transaction is matching more with legal pattern of the particular customer, then the algorithm returns “0” (i.e., legal transaction) and if the incoming transaction is matching more with fraud pattern of that customer, then the algorithm returns “1” (i.e., fraudulent transaction).

#### 2.5.1. Pattern Database Construction Using Frequent Itemset Mining (Training)

Frequent itemsets are sets of items that occur simultaneously in as many transactions as the user defined minimum support. The metric support(*X*) is defined as the fraction of records of database *D* that contains the itemset *X* as a subset:

(1) $$\begin{array}{c} \text{support}\left(X\right)=\frac{\text{count}\left(X\right)}{\left|D\right|}. \end{array}$$

For example, if the database contains 1000 records and the itemset *X* appears in 800 records, then the support(*X*) = 800/1000 = 0.8 = 80%; that is, 80% of transactions support the itemset *X*.

In credit card transaction data, the legal pattern of a customer is the set of attribute values specific to a customer when he does a legal transaction which shows the customer behavior. It is found that the fraudsters are also behaving almost in the same manner as that of a customer [1]. This means that fraudsters are intruding into customer accounts after learning their genuine behavior only. Therefore, instead of finding a common pattern for fraudster behavior it is more valid to identify fraud patterns for each customer. Thus, in this research, we have constructed two patterns for each customer—legal pattern and fraud pattern. When frequent pattern mining is applied to credit card transaction data of a particular customer, it returns set of attributes showing same values in a group of transactions specified by the support. Generally the frequent pattern mining algorithms like that of Apriori [31] return many such groups and the longest group containing maximum number of attributes is selected as that particular customer's legal pattern. The training (pattern recognition) algorithm is given below.

**Step 1 .**

Separate each customer's transactions from the whole transaction database *D*.

**Step 2 .**

From each customer's transactions separate his/her legal and fraud transactions.

**Step 3 .**

Apply Apriori algorithm to the set of legal transactions of each customer. The Apriori algorithm returns a set of frequent itemsets. Take the largest frequent itemset as the legal pattern corresponding to that customer. Store these legal patterns in legal pattern database.

**Step 4 .**

Apply Apriori algorithm to the set of fraud transactions of each customer. The Apriori algorithm returns a set of frequent itemsets. Take the largest frequent itemset as the fraud pattern corresponding to that customer. Store these fraud patterns in fraud pattern database.

The pseudocode of training algorithm is given in Algorithm 1.

#### 2.5.2. Fraud Detection Using Matching Algorithm (Testing)

After finding the legal and fraud patterns for each customer, the fraud detection system traverses these fraud and legal pattern databases in order to detect frauds. These pattern databases are much smaller in size than original customer transaction databases as they contain only one record corresponding to a customer. This research proposes a matching algorithm which traverses the pattern databases for a match with the incoming transaction to detect fraud. If a closer match is found with legal pattern of the corresponding customer, then the matching algorithm returns “0” giving a green signal to the bank for allowing the transaction. If a closer match is found with fraud pattern of the corresponding customer, then the matching algorithm returns “1” giving an alarm to the bank for stopping the transaction. The size of pattern databases is *n* × *k* where *n* is the number of customers and *k* is the number of attributes. The matching (testing) algorithm is explained below.

**Step 1 .**

Count the number of attributes in the incoming transaction matching with that of the legal pattern of the corresponding customer. Let it be *lc*.

**Step 2 .**

Count the number of attributes in the incoming transaction matching with that of the fraud pattern of the corresponding customer. Let it be *fc*.

**Step 3 .**

If *fc* = 0 and *lc* is more than the user defined matching percentage, then the incoming transaction is legal.

**Step 4 .**

If *lc* = 0 and *fc* is more than the user defined matching percentage, then the incoming transaction is fraud.

**Step 5 .**

If both *fc* and *lc* are greater than zero and *fc* ≥ *lc*, then the incoming transaction is fraud or else it is legal.

The pseudocode of the testing algorithm is given in Algorithm 2.

## 3. Experiment

The experiment was carried out in Intel Core i5 processor with 4 GB RAM. Both training and testing algorithms are implemented in MATLAB R2013a.

### 3.1. UCSD-FICO Data Mining Contest 2009 Dataset

In order to evaluate the proposed model, UCSD-FICO Data mining contest 2009 data set is used. The competition was organized by FICO, the leading provider of analytics and decision management technology, and the University of California, San Diego (UCSD). The dataset is a real dataset of e-commerce transactions and the objective was to detect anomalous e-commerce transactions. They provided two versions of the dataset: “easy” and “hard” versions and we have used the “hard” version for the evaluation of our model. Moreover, the fields of the dataset are anonymized so strongly that it is hard to derive any new field and thus the fraud detection methods depending on aggregation and derived attributes will not work efficiently on this data.

### 3.2. Data Preprocessing

The hard version of the dataset contains two sub datasets—training set and testing set. The training set is labeled and the testing set is unlabeled. We have used only the labeled training dataset. It contains 100000 transactions of 73729 customers spanning over a period of 98 days. The dataset contains 20 fields including class labels—amount, hour1, state1, zip1, custAttr1, field1, custAttr2, field2, hour2, flag1, total, field3, field4, indicator1, indicator2, flag2, flag3, flag4, flag5, and Class. It is found that* custAttr1* is the account/card number and* custAttr2* is e-mail id of the customer. Both these fields are unique to a particular customer and thus we decided to keep only* custAttr1*. The fields* total *and* amount* as well as* hour1* and* hour2* are found to be the same for each customer and thus we removed* total* and* hour2*. Similarly* state1* and* zip1* are also found to be representing the same information and thus we removed* state1*. All other fields are anonymized and therefore we decided to keep them as they are. Thus our final dataset contains 16 fields—amount, hour1, zip1, custAttr1, field1, field2, flag1, field3, field4, indicator1, indicator2, flag2, flag3, flag4, flag5, and Class.

### 3.3. Training and Testing Dataset Creation

The following procedures are used for creating training and testing datasets for evaluating our model.

1. First, we removed the transactions corresponding to those customers who have only one transaction in dataset since it appears either in training or testing dataset only. Now the dataset has been reduced to 40918 transactions.
2. Then we divided these 40918 transactions into two sets—training set with 21000 transactions and testing set with 19918 transactions.
3. Again from the training dataset we removed the transactions corresponding to those customers who have only one transaction in the training dataset since it is hard to find a pattern from a single transaction. Now the training dataset has been reduced to 19165 transactions.
4. From this dataset, we have randomly selected different groups of customers and their corresponding transactions in the training and testing dataset to create different training and testing datasets to evaluate the performance of FraudMiner with increasing number of transactions. The data distribution is shown in Table 1.

### 3.4. Legal/Fraud Pattern Creation

From the training set (for each group) in Table 1, fraud and legal patterns are created for each customer by using the proposed training algorithm. We set the minimum support as 0.9 and selected the large itemset as the pattern.

For example, let the largest itemset be

 
*hour = 0 *
 
*zip = 950 *
 
*field1 = 3 *
 
*field2 = 0 *
 
*field3 = 2429 *
 
*field4 = 14 *
 
*indicator1 = 0 *
 
*indicator2 = 0 *
 
*flag1 = 0 *
 
*flag2 = 0 *
 
*flag3 = 0 *
 
*flag4 = 0 *
 
*flag5 = 1.*

Then the corresponding pattern is

(2) 0 ∣ 9999 ∣ 950 ∣ 3 ∣ 0 ∣ 2429 ∣ 14 ∣ 0 ∣ 0 ∣ 0 ∣ 0 ∣ 0 ∣ 0 ∣ 1

Here* “9999”* represents an invalid field because this field has different values in each transaction and hence it is not contributing to the pattern.

## 4. Results

The performance of the proposed classifier is evaluated in terms of 4 classification metrics (http://www.damienfrancois.be/blog/files/modelperfcheatsheet.pdf) relevant to credit card fraud detection—fraud detection rate, false alarm rate, balanced classification rate, and Matthews correlation coefficient. The other common metrics like accuracy and error rate are known to be bias metrics in the case of imbalance and hence we did not consider them. Here, fraud is considered as positive class and legal as negative class and hence the meaning of the terms P, N, TP, TN, FP, and FN are defined as follows:

 positives (P): number of fraud transactions;
 negatives (N): number of legal transactions;
 true positives (TP): number of fraud transactions predicted as fraud;
 true negatives (TN): number of legal transactions predicted as legal;
 false positives (FP): number of legal transactions predicted as fraud;
 false negatives (FN): number of fraud transactions predicted as legal.

Also the performance of the proposed fraud detection model (FraudMiner) is compared with 4 other states of the art classifiers used for credit card fraud detection [10]: support vector machine (SVM) [27], K-nearest neighbor classifier [28], naïve Bayes classifier [29], and random forest [30]. These are the base classifiers used in the state-of-the-art financial fraud detection models described in the literature review. Among these classifiers random forest is used by the winner of the UCSD-FICO Data Mining Contest 2009 (http://www.quansun.com/ucsd-data-mining-contests.htm). We tried to apply SMOTE [32], an oversampling technique, that used common-to-handle class imbalance, before giving the data to the classifiers. But the performance of the classifiers is found degrading because of the highly imbalanced nature of the dataset. Hence we supplied the data directly to the classifiers.

### 4.1. Sensitivity/Fraud Catching Rate

Sensitivity represents the portion of actual positives which are predicted positives. In credit card fraud detection, sensitivity denotes the fraud detection rate and it is defined as

(3) $$\begin{array}{c} \text{Sensitivity}=\frac{\text{TP}}{\text{P}}. \end{array}$$

**Figure 2:**
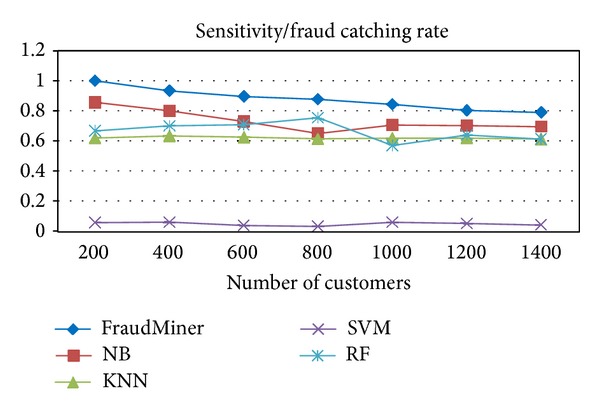
Performance comparison of classifiers on sensitivity.

Figure 2 shows the performance of FraudMiner on* sensitivity* in comparison with other classifiers.

### 4.2. False Alarm Rate

False alarm rate represents portion of actual negatives which are predicted as positives and it is defined as

(4) $$\begin{array}{c} \text{False}\;\;\text{Alarm}\;\;\text{Rate}=\frac{\text{FP}}{\text{N}}. \end{array}$$

**Figure 3:**
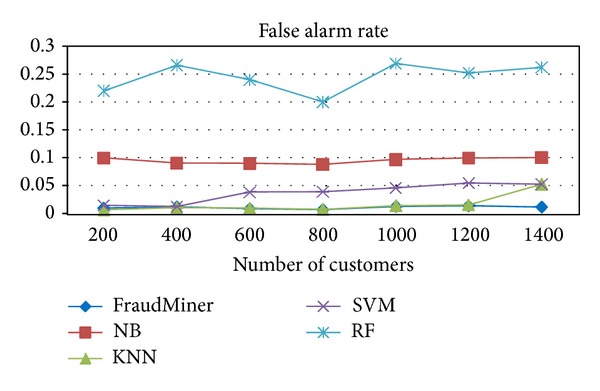
Performance comparison of classifiers on false alarm rate.

Figure 3 shows the performance of FraudMiner on* false alarm rate* in comparison with other classifiers. This metric should be low since false alarm leads to customer dissatisfaction.

### 4.3. Balanced Classification Rate (BCR)

Balanced classification rate is the average of sensitivity and specificity and is defined as

(5) $$\begin{array}{c} \text{BCR}=\frac{\text{TP}}{\text{P}}+\frac{\text{TN}}{\text{N}}. \end{array}$$

**Figure 4:**
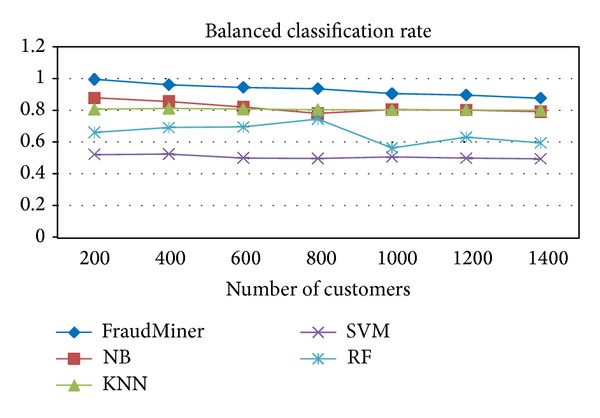
Performance comparison of classifiers on balanced classification rate.

Figure 4 shows the performance of FraudMiner on* balanced classification rate* in comparison with other classifiers.

### 4.4. Matthews Correlation Coefficient (MCC)

Matthews correlation coefficient is used as a measure of the quality of binary classifications. It takes into account true and false positives and negatives and is a balanced measure which can be used in imbalanced data like credit card transaction data. The MCC is a correlation coefficient between the observed and predicted binary classifications and its value is between −1 and +1. A coefficient of +1 represents a perfect prediction, 0 no better than random prediction, and −1 indicates total disagreement between prediction and observation. It is defined as

(6) $$\begin{array}{c} \text{MCC}=\frac{\left(\text{TP}*\text{TN}\right)-\left(\text{FP}*\text{FN}\right)}{\sqrt{\left(\text{TP}+\text{FP})\left(\text{TP}+\text{FN}\right)\left(\text{TN}+\text{FP}\right)(\text{TN}+\text{FN}\right)}}. \end{array}$$

**Figure 5:**
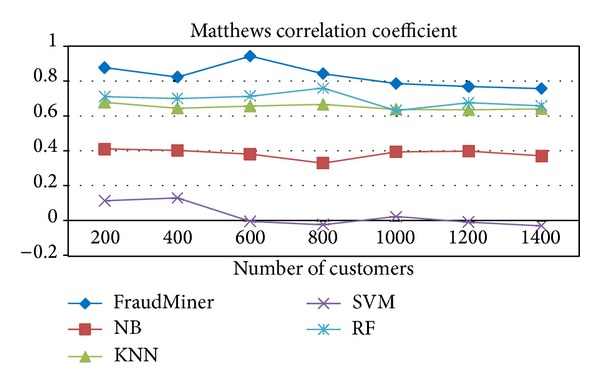
Performance comparison of classifiers on MCC.

Figure 5 shows the performance of FraudMiner on* Matthews correlation coefficient* in comparison with other classifiers.

## 5. Discussion

In fraud detection, the most important measure is sensitivity or fraud detection rate, since the loss due to fraud depends on this metric. From the performance evaluation it is found that FraudMiner is having the* highest fraud detection rate* (Figure 2) than other classifiers. The second important measure is the* false alarm rate*, since it shows the customer dissatisfaction due to false alarm (legal transaction, but suspected as fraud). FraudMiner shows very less false alarm rate (Figure 3).

Two* balanced metrics, BCR *and* MCC, *are used to evaluate the capability of FraudMiner for handling class imbalance and FraudMiner showed very good performance according to these measures compared with other classifiers (Figures 4 and 5).

It is found that our model could not recognize only those frauds, where there is no pattern difference between the legal and fraud transactions (overlapping). For example, consider the two transactions in the test data set shown in Table 2.

Here the attributes of both transactions are the same. But one is legal and the other is fraud. FraudMiner could not recognize this fraud transaction because the pattern database contains only legal pattern for this customer and both transactions are matching with that pattern. It is found that when both fraud and legal patterns for a customer are available in the pattern database, then FraudMiner shows 100% fraud detection capability.

## 6. Conclusion

This paper proposes a fraud detection model whose performance is evaluated with an anonymized dataset and it is found that the proposed model works well with this kind of data since it is independent of attribute values. The second feature of the proposed model is its ability to handle class imbalance. This is incorporated in the model by creating two separate pattern databases for fraud and legal transactions. Both customer and fraudulent behaviors are found to be changing gradually over a longer period of time. This may degrade the performance of fraud detection model. Therefore the fraud detection model should be adaptive to these behavioral changes. These behavioral changes can be incorporated into the proposed model by updating the fraud and legal pattern databases. This can be done by running the proposed pattern recognition algorithm at fixed time points like once in 3 months or six months or once in every one lakh transaction. Moreover the proposed fraud detection method takes very less time, which is also an important parameter of this real time application, because the fraud detection is done by traversing the smaller pattern databases rather than the large transaction database.

## Figures and Tables

**Algorithm 1:**
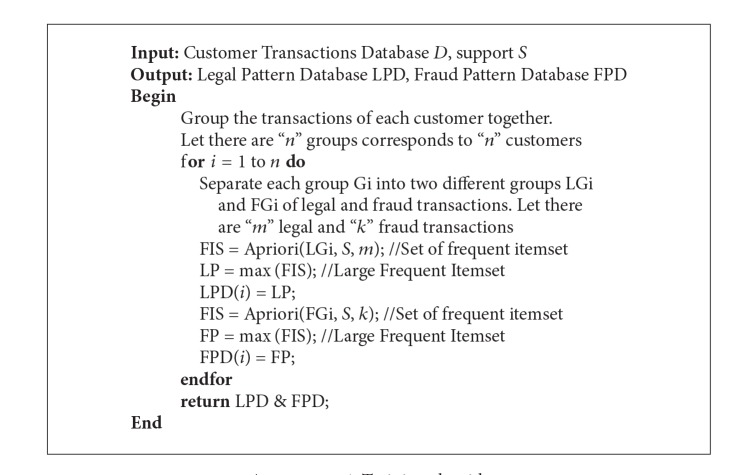
Training algorithm.

**Algorithm 2:**
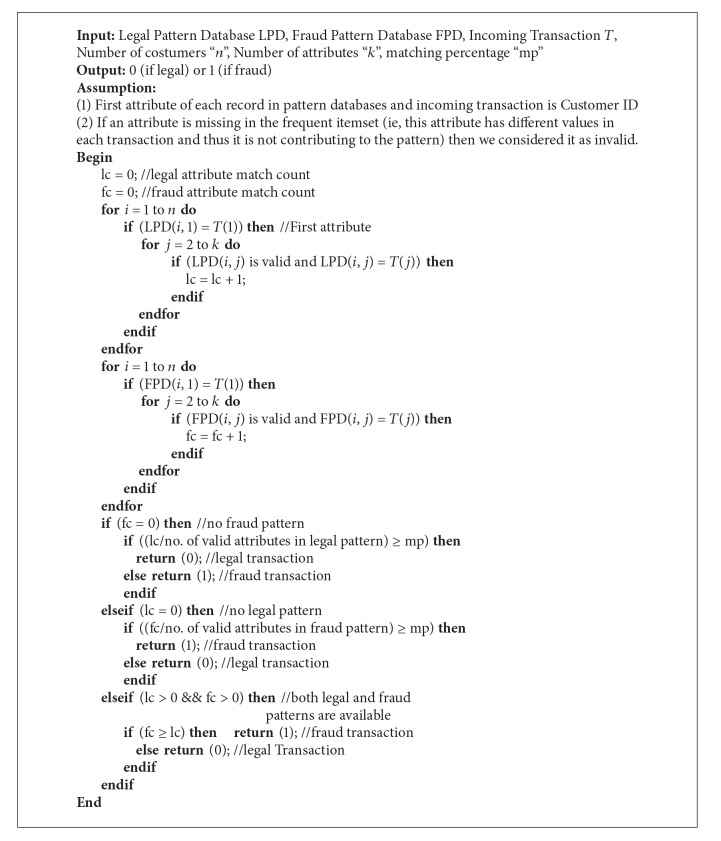
Testing algorithm.

**Table 1:** Imbalanced data.

Number of customers	Number of transactions in training set			Number of transactions in testing set		
	Legal	Fraud	Total	Legal	Fraud	Total
200	652	25	677	489	17	506
400	1226	48	1274	864	30	894
600	1716	64	1780	1244	48	1292
800	2169	71	2240	1612	57	1669
1000	2604	131	2735	2002	102	2104
1200	3056	157	3113	2604	144	2748
1400	3440	158	3598	3083	147	3230

**Table 2:** Overlapped dataset.

custAttr1	amount	hour1	zip1	field1	field2	fielsd3	field4	indicator1	indicator2	flag1	flag2	flag3	flag4	flag5	Class
1234567890123867	12.95	9	432	3	0	5454	10	0	0	1	0	0	0	1	1
1234567890123867	12.95	9	432	3	0	5454	10	0	0	1	0	0	0	1	0
